# Survey on Energy Harvesting for Biomedical Devices: Applications, Challenges and Future Prospects for African Countries

**DOI:** 10.3390/s24010163

**Published:** 2023-12-27

**Authors:** Djakou Nekui Olivier, Wei Wang, Cheng Liu, Zhixia Wang, Bei Ding

**Affiliations:** Tianjin Key Laboratory of Nonlinear Dynamics and Control, School of Mechanical Engineering, Tianjin University, Tianjin 300350, China; olivierdjakou@tju.edu.cn (D.N.O.); liucheng94@tju.edu.cn (C.L.); zhixiawang@tju.edu.cn (Z.W.); dingbei_784@tju.edu.cn (B.D.)

**Keywords:** Africa, biomedical research, biomedical research funding, energy harvesting, self-powered biomedical devices

## Abstract

Self-powered biomedical devices, which are the new vision of Internet Of Things (IOT) healthcare, are facing many technical and application challenges. Many research works have reported biomedical devices and self-powered applications for healthcare, along with various strategies to improve the monitoring time of self-powered devices or to eliminate the dependence on electrochemical batteries. However, none of these works have especially assessed the development and application of healthcare devices in an African context. This article provides a comprehensive review of self-powered devices in the biomedical research field, introduces their applications for healthcare, evaluates their status in Africa by providing a thorough review of existing biomedical device initiatives and available financial and scientific cooperation institutions in Africa for the biomedical research field, and highlights general challenges for implementing self-powered biomedical devices and particular challenges related to developing countries. The future perspectives of the aforementioned research field are provided, as well as an architecture for improving this research field in developing countries.

## 1. Introduction and Theoretical Background

Energy harvesting (EH) technologies, the future of wearable devices, provide promising solutions to overcome the short lifetimes of wearable devices. In recent decades, modern wearable technologies, including biosensors, wearable fitness trackers (WFTs), smart health watches, wearable electrocardiogram (ECG) monitors, wearable blood pressure monitors, continuous glucose meters, etc., have widely impacted our lifestyle. According to GlobalData [1], the wearable technology market was estimated to be approximately USD 46 billion in 2022 and is forecast to grow to over USD 100 billion by 2027, with a compound annual growth (CAGR) of 17%. 

The key factors driving the wearable technology market growth include the increasing popularity of the Internet of Things (IoT) and connected devices, device miniaturization for wearability, increasing demand for wearable devices for monitoring and tracking health vital signs, and rapid advancements in sensor technology. 

Self-powered technology means that a device can sustain its own operation by harvesting power from its working environment without an external power supply. Other definitions of self-powered devices from different organizations are summarized in Table 1.

Given the significant, widespread applications of the aforementioned biomedical devices, many concerns have been raised about the weight and the availability of power sources for wearable devices. In fact, batteries developed for wearable applications have contributed to the achievement of successful deployment in healthcare [3,4]. The implanted devices are meant to continually assess patient health on a predetermined scheme, which constrains the designers of biomedical applications, requiring long-life batteries to be chosen to avoid frequent replacement. In addition, batteries must have a volumetric high energy density to enable the design of miniaturized implants and avoid discomfort and harm to patients [5,6]. In an attempt to overcome the limitations of traditional batteries in wearable applications, there have been undertakings for developing wearable devices with non-exogenous power requirements. Electrochemical cells and various transduction techniques have been introduced as implantable power sources [7,8]. Their short shelf life, the voltage and current instability, and the presence of hydrogen gas limit their application. Devices such as piezoelectric materials have been introduced to continuously recharge the batteries of pacemakers by the direct conversion of heartbeats to electric energy. Even though piezoelectric transduction techniques have contributed to the achievement of effective self-powered pacemakers, they are also an invasive solution. In fact, in comparison with traditional pacemakers, requiring frequent surgery to charge the battery of the device, self-powered pacemakers require surgery after a long period only for replacing the battery of the implanted device [9]. Hanjun Ryu et al., have developed a commercial self-rechargeable cardiac pacemaker system with an implanted inertia-driven triboelectric nanogenerator (I-TENG) based on body motion and gravity, which has the potential to extend the device operating time and reduce risks of regular surgery [10]. Radio imaging has been used for many years to evaluate bone healing after surgery. The side effect of radio imaging is the exposure of patients to radiation, which damages deoxyribonucleic acid (DNA) in patient cells and causes cancer in the long run [11,12]. In order to overcome the shortcomings of radio imaging, a reliable solution was introduced by Amir Alavi et al. The research team designed a smart, self-aware implant for tracking spinal fusion for bone healing. The meta-triboelectric material is inserted into inter-vertebral discs, and during bone healing, the load applied on the discs reduces gradually. The applied load on the meta-triboelectric material generates electric energy, which is used to assess bone healing [13]. 

The research and development of self-powered biomedical devices in Africa is still at its early stage. Further development is provided in Section 3, along with suggestions for improving the research field. However, considerable achievements by African developers for local healthcare have been reported. The CARDIOPAD device developed by Cameroonian Arthur Zang enables the monitoring of heartbeat rates and forwards data to a remote scientist or cardiology hospital for diagnosis [14]. Tiam Kapen P. et al., have reported a multi-function neonatal incubator for low- and middle-income countries [15]. Many other biomedical devices that can be used in Africa healthcare have been developed, such as a blood glucose meter in Africa for Africans [16]; a smart, low-cost, non-invasive blood glucose monitoring device in South Africa [17], a free play fetal heart rate monitor [18], the SINAPI chest drain [19], etc.

The potential energy sources for deployable devices, such as solar energy, chemical energy, electromechanical energy and thermal energy, are under intensive investigation. Electromagnetic energy is derived from the motion of a coil trough a stationary field [20,21], triboelectricity is generated from friction between two different materials [10], and piezoelectric energy results from the deformation of a piezoelectric material [22,23]. The above transducers are capable of converting primary energy sources available in human environments to electric energy. Therefore, triboelectric, electromagnetic and piezoelectric energy is suitable for self-powered biomedical devices due to the possibility to miniaturize their design and achieve a volumetric high energy density.

Given the promising opportunities of bio-mechanical energy harvesting for self-powered devices in global healthcare, the leading questions for this review are:

▪ How can free and available energies in the human environment be turned into a power source for embedded healthcare devices?
▪ What are the challenges and opportunities?
▪ Are African countries ready for facing the challenges, or are there any findings in developing countries in similar topics for local healthcare?

The ideal way to obtain a clear answer to these questions is by providing a thorough review of self-powered biomedical devices developed so far for healthcare, highlighting the challenges and opportunities for their application in an African context and demonstrating some achievements in similar topics in Africa for local healthcare. This will be achieved by following the research string provided in [24], which conforms with the Preferred Reporting Items for Systematic Reviews and Meta-analysis (PRISMA) guidelines.

## 2. Review Methodology

A research framework is provided for designing, conducting and analyzing review articles systematically and rigorously. It is helpful for achieving review consistency.

### 2.1. Systematic Literature Review (SLR)

The systematic literature review provided in [24] has been shortened into five (05) main steps. Some steps are merged into others to reduce the length of the review.

#### 2.1.1. Purpose of the Literature Review

The purpose of this literature review is to highlight the achievements of bio-mechanical energy harvesting for self-powered healthcare devices, outline the current state of progress of some African countries in the aforementioned research field by presenting biomedical research and devices initiatives developed so far for solving local health issues, point out the challenges and opportunities, and provide objective advice to determine the implementation challenges of biomedical research in developing countries.

#### 2.1.2. Protocol and Training

**Figure 1:**
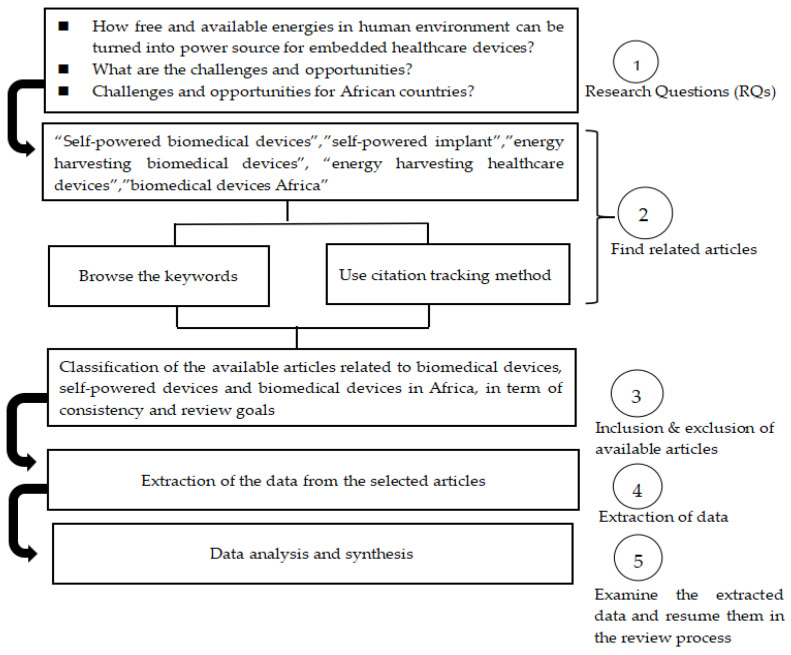
Systematic review protocol followed for answering the research questions (RQs).

A review protocol is designed in Figure 1 for ensuring the review consistency and answering the research questions (RQs).

#### 2.1.3. Screening of the Existing Literature

The following search strings were defined “self-powered biomedical devices”, “self-powered implant”, “energy-harvesting biomedical devices”, “energy-harvesting healthcare devices”, and “biomedical devices Africa”. Besides the predefined search strings, the pearl growing or citation tracking method was used in order not to rely only on a protocol-driven strategy and miss other important resources.

#### 2.1.4. Extraction and Appraisal of Data Quality

The criteria considered for the selection of studies include:

▪ Articles written in English and published within eight recent years (2015–2023);
▪ Applied research and technology development articles;
▪ Articles related to biomedical energy harvesting and healthcare devices.

A total of 54 research articles summarized in Table 2 and Table 3 were included for further review.

The remaining articles were excluded.

Some important articles might have been excluded because their publication date was out of the selected review range or because their content does not include both aspects of energy harvesting and biomedical devices. In addition, articles including experimental studies were the priority of this review.

#### 2.1.5. Study Synthesis

**Figure 2:**
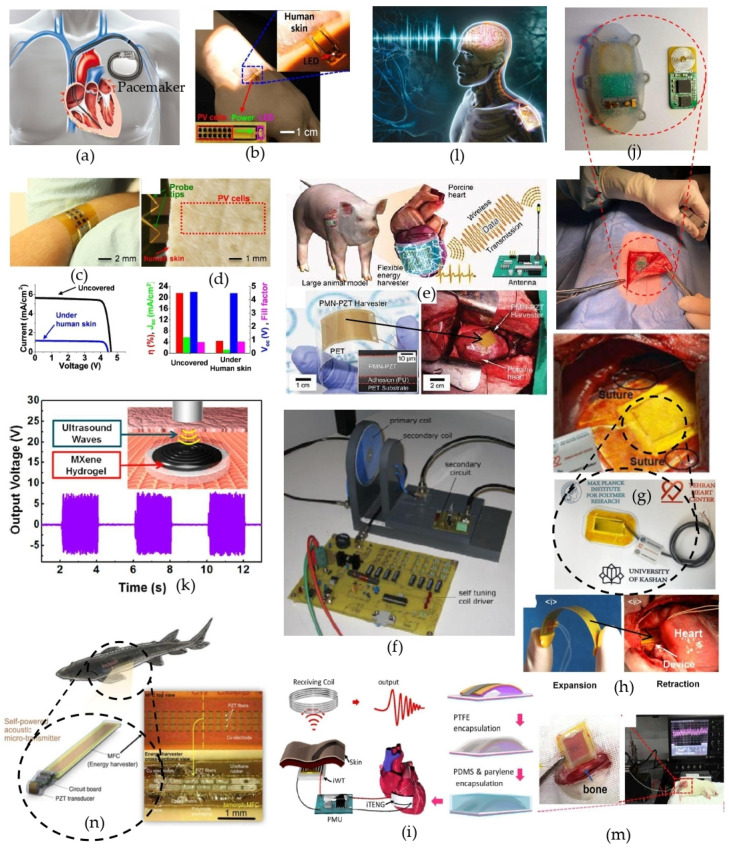
Illustration of the potential applications of implanted PV devices for powering implantable electronics such as pacemakers (**a**). The feasibility of the study is shown by lighting LEDs with power from integrated PV devices under human hand dorsum skin (**b**). Optical image of IPV device bent on a human arm (**c**), image of fixed human skin covering IPV cells (**d**) [25]. In vivo self-powered cardiac sensor for estimating blood pressure and velocity of blood flow (**e**) [26]. Self-tuning inductive powering system for biomedical implants (**f**) [27]. Self-powered cardiac pacemaker with a piezoelectric polymer nanogenerator implant (**g**) [28]. Implantable and self-powered blood pressure monitoring based on a piezoelectric thin film (**h**) [29]. Schematic diagram of a self-powered wireless transmission system based on an implanted triboelectric nanogenerator (iWT: implantable Wireless Transmitter; PMU: Power Management Unit) (**i**) [30]. A battery-less implantable glucose sensor based on electrical impedance spectroscopy; sensor implantation on the pig for experimentation (**j**) [31]. Biocompatible battery for medical implant charged via ultrasound (**k**) [32]. Self-powered deep brain stimulation via a flexible PIMNT energy harvester (**l**) [33]. Self-powered implantable electrical stimulator for osteoblast proliferation and differentiation (**m**) [34]. An implantable biomechanical energy harvester for animal monitoring devices (**n**) [35]. Reproduced with permission from [25,26,27,28,29,30,31,32,33,34,35].

**Figure 3:**
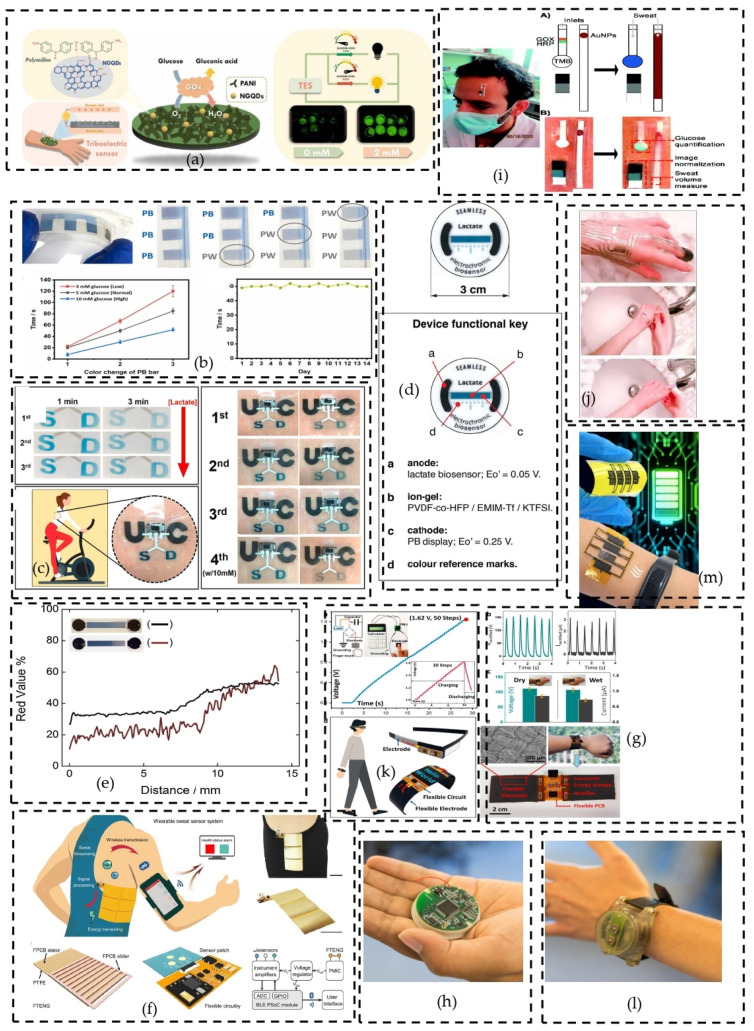
Schematic illustration of the potential applications of non-invasive, wearable, self-powered devices. Non-invasive glucose meters (**a**–**e**) [36,37,38,39,40]. Wireless, battery-free wearable sweat sensor powered by human motion, along with the schematic illustrating human motion energy harvesting, signal processing, microfluidic-based sweat biosensing, and Bluetooth-based wireless data transmission to a mobile user interface for real-time health status tracking (**f**) [41]. Wearable applications of body-integrated self-powered systems (BISSs) (**g**) [42]. Behavioral and environmental sensing and intervention (BESI), which combines environmental sensors placed around the homes of dementia patients for detecting the early stage of agitation (**h**) [43]. Schematic representation of glucose level detection in human sweet (**i**) [44]. Wearable circuits sintered at room temperature directly on the skin surface for health monitoring (**j**) [45]. Diagram of flexible, wearable, self-powered electronics based on a body-integrated self-powered system (BISS) (**k**) [42]. Technology-Enabled Medical Precision Observation (TEMPO): a wristwatch-sized device that can be worn on various parts of the body for monitoring user’s agitation during motion and detect early cerebral palsy, Parkinson’s disease and multiple sclerosis (**l**) [43]. The device was developed by the University of Virginia. Stretchable micro-supercapacitors which harvest energy from human breathing and motion for self-powering wearable devices (**m**) [46]. Reproduced with permission from [36,37,38,39,40,41,42,43,44,45,46].

Following the research questions introduced earlier, and based on the review articles found during systematic research review, it is clear that the contribution of biomedical energy harvesting for self-powered devices is undeniable in human healthcare. Evidence includes invasive self-powered biomedical devices, summarized in Figure 2, and non-invasive self-powered devices, summarized in Figure 3.

▪ Invasive self-powered biomedical devices.

Biomedical electronics powered by solar cells were developed by Song K. et al. [25]. Figure 2a–d illustrate the potential applications of implanted PV devices for powering biomedical implants such as pacemakers. A miniaturized implanted photovoltaic cell (IPV) is inserted under small top layer of human skin (0.68 mm thickness) for harvesting electric energy and powering biomedical implants such as pacemakers, etc., which enables avoiding regular surgery for replacing the batteries of biomedical implants powered by traditional batteries.

Various harvesting strategies have been implemented by researchers for self-powered implantable biomedical and tracking devices, including an in vivo self-powered cardiac sensor for estimating blood pressure and the velocity of blood flow [26], a self-tuning inductive powering system [27], a bio-compatible, flexible piezoelectric polymer-based nanogenerator for powering cardiac pacemakers (PNG) [28], and piezoelectric thin film (PETF)-based energy harvesting [29], which harvests energy from the axial stress of artery to measure blood pressure. An in vivo self-powered cardiac sensor was developed for estimating blood pressure and the velocity of blood flow [30]. A battery-less implantable glucose sensor based on electrical impedance spectroscopy was developed for continuous monitoring of blood glucose concentrations [31]. An ultrasound-driven two-dimensional Ti3C2Tx MXene hydrogel generator, which harvests ambient vibrations coupled with triboelectrification, for powering implanted generators was proposed [32]. Self-powered deep brain stimulation via a flexible point energy harvester, which has the potential to generate electricity from cyclic deformations of the heart, lungs, muscles, and joints, was proposed to supply electric energy to a deep brain stimulation system and induce behavioral changes in a living body [33]. A self-powered flexible and implantable electrical stimulator was developed, which consists of a triboelectric nanogenerator (TENG) and a flexible interdigitated electrode for osteoblast proliferation and differentiation [34]. A Macro Fiber Composite (MFC) piezoelectric beam for harvesting bending movement from fishes for powering tracking devices was also developed [35], among others.

▪ Non-invasive self-powered biomedical devices.

Besides implantable devices, non-invasive wearable biomedical energy harvesting systems for biosensors have drawn the attention of many researchers. Some applications include non-invasive wearable self-powered triboelectric sensors (TESs) for simultaneous physiological monitoring developed by Yu-Hsin Chang et al., which enables the assessment of glucose concentrations in human sweat. The triboelectric sensing layer is made of nanocomposite N-doped graphene quantum-dot-decorated polyaniline (NGQDs/PANI)/Glucose oxidase enzine (GOx) for non-invasive monitoring of glucose levels in human sweat, as shown in Figure 3a [36]. An electric voltage is generated due to the coupling effect of enzymatic reaction and triboelectrification. The glucose concentration is proportional to the generated voltage. LEDs are used as an indicator of glucose concentrations. Figure 3b shows a flexible, disposable and self-powered glucose biosensor visible to the naked eye developed by J. Lee et al. [37], in which the theory of enzymatic biofuel cells is adopted for self-power generation. The polarization of the glucose biosensor enables the Prussian blue (PB) bar in Figure 3b to automatically change color, which is used to indicate three levels of glucose, including low, normal and high glucose concentrations, in human sweat. Many other non-invasive self-powered biosensors for glucose level assessments have been developed based on the same principle of enzymatic reactions, such as a resettable sweat-powered wearable electrochromic biosensor (Figure 3c) by M.C. Hartel et al. [38], a self-powered skin-patch electrochromic biosensor (Figure 3d) by S. Santiago-Malagon et al. [39], and fully printed and silicon-free self-powered electrochromic biosensors for naked eye quantification (Figure 3e) developed by M. Aller-Pellitero et al. [40].

Non-invasive glucose detection strategies have shown the efficiency of glucose meters without an external power supply.

▪ Comparison of biomedical devices powered by nanogenerators

A comparison of self-powered biomedical devices is provided in Table 3. Self-powered systems harvesting energy from photovoltaic solar cells can achieve the maximum power density. They are best suited to self-powered non-invasive biomedical applications, since photovoltaic solar cells can be exposed to sunlight. However, for invasive biomedical self-powered applications, triboelectric energy generation enables harvesting the maximum power for the smallest design surface.

## 3. Challenges, Opportunities, Status and Capability of Self-Powered Biomedical Devices

### 3.1. Challenges of Self-Powered Biomedical Devices

Challenges for implementing self-powered biomedical devices in an Africa context are categorized into general challenges for self-powered bio-electronics and specific challenges for developing countries.

**Figure 4:**
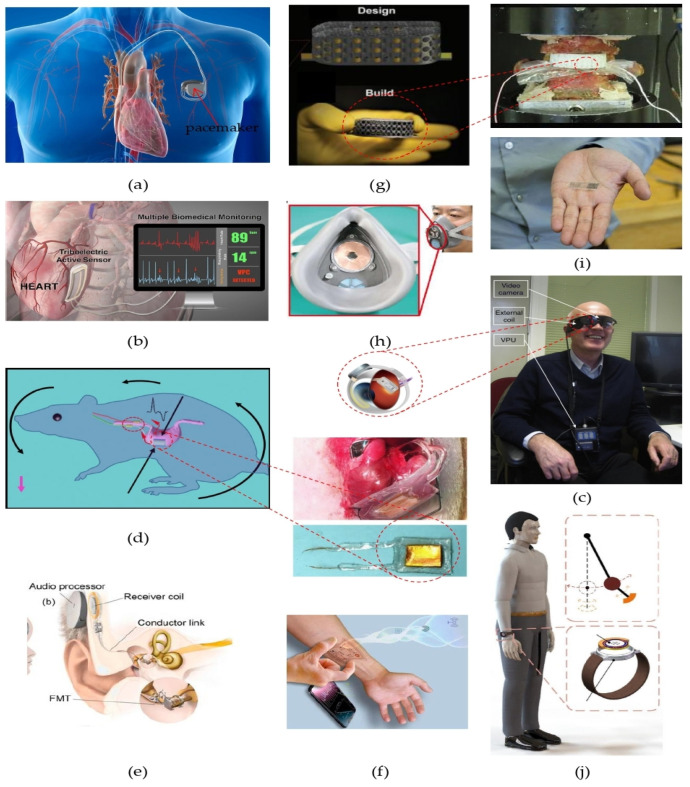
Examples of miniaturized biomedical devices and self-powered implants. Self-rechargeable cardiac pacemaker (**a**) [72]; troboelectric active sensor (**b**) [72,73]; retinal prosthesis system, a variable external unit with camera attached to it (**c**) [74]; self-powered vagus nerve stimulator device for effective weight control (**d**) [75]; an ultrasonic energy harvester in use in a cochlear hearing aid (**e**) [76]; energy harvesting from radio waves for powering wearable devices (**f**) [77]; self-powered metamaterial implant for the detection of bone healing progress (**g**) [13]; self-powered electrostatic adsorption face mask based on a triboelectric nanogenerator (**h**) [78]; self-powered implantable device for stimulating fast bone healing, which then disappears without a trace (**i**); self-powered smart watch and wristband enabled by an embedded generator (**j**) [79]. Reproduced with permission from [13,72,73,74,75,76,77,78,79].

Self-powered healthcare devices face general challenges such as design constraints, the comfort and safety of patients [71]. In fact, self-powered biomedical devices aim to achieve compatibility with living tissues and organs, in vivo stability, design miniaturization and low power consumption. Figure 4 shows several examples of biomedical devices for in vivo and non-invasive healthcare applications to illustrate the complexity of the devices.

▪ Challenge of miniaturization: The design aims to miniaturize the size and achieve the highest output power performance, which are contradictory;
▪ Flexibility: In vivo self-powered systems need the highest flexibility in order to not harm patients;
▪ Biocompatibility: Biomedical devices must meet the appropriate biological requirements for a biomaterials;
▪ Long-term stability: Biomedical devices must be able to operate for a long period, especially for in vivo applications, in order to avoid regular surgery.

Specific challenges for implementing self-powered devices in developing countries include insufficient digital literacy training, infrastructure, local Artificial Intelligence (AI) talents, data sets and government support.

▪ Insufficient Digital literacy training and infrastructures.

Enduring lessons from the COVID-19 crisis have shown how sustainable development in global healthcare can only be achieved when no one is left behind. They have shown the importance of the availability of digital infrastructures for remote healthcare, efficient management of quarantined patients, etc.

Compared to other continents, digital penetration in Africa is still low in spite of remarkable achievements by young talent [14,15,16,17,18,19,47,48,49,50,51,52,53,54,55,56] and efforts from governments and non-profit organizations (NPOs).

Barriers for digital literacy growth in Africa include inability to afford training and experimental infrastructure and insufficient information (many IoT developers are unaware of recent trends in biomedical devices and their contribution in healthcare). Political instabilities, which lead to armed conflicts and inaccessibility to education in some countries are also a barrier. Electricity and internet supplies are fragmented in many rural areas in Africa. According to a World Bank report, less than 51% of the rural area population in sub-Sahara Africa had access to electricity in 2021 [80].

▪ Data sets and government support.

Another major issue faced by the development of biomedical devices in Africa is the lack of accessible data to African AI talents and the relevance of the available data to local problems. In fact, machine learning applications, such as voice and pattern recognition, rely on a large amount of data for testing algorithms. Available data are collected from patients with different ethnicities and from different environments. Inappropriate data or their misinterpretation might be harmful, especially when it comes to healthcare applications.

Artificial Intelligence (AI), especially in the biomedical research field, faces the common problem of insufficient support from African governments as well. In the European Union and North America, governments have set rules governing the use and application of Artificial Intelligence for personal and commercial uses. Sub-Saharan African countries have been classified as the worst-performing region in the 2023 Government AI Readiness Index, which emphasizes the serious challenges to AI adoption in Africa.

Despite these barriers, there have been significant improvements in some countries such as Rwanda, Senegal and Benin in setting a new framework for new national AI strategies and announcing these forthcoming strategies. Mauritius remains the only country in the region with an AI strategy over the past 5 years [81].

### 3.2. Opportunities for African Countries

The World Health Organization (WHO) has reported that cardiovascular diseases (CVDs) are the first cause of death globally. Out of 17 million premature deaths (under the age of 70) due to non-communicable diseases in 2015, 82% were from low- and middle-income countries and 37% were caused by CVDs [82]. Sufficient support for new infrastructures, data sets and AI regulations from governments, as mentioned in the previous section, can enable the development of self-powered microelectronic devices relevant to healthcare in an African context for Africans.

Self-powered devices show high potential in energy harvesting, sensing, healthcare and biomedical implants and are becoming a new area of electronics devices in IOT applications. Self-powered devices improve the existing systems by enabling the device to supply its own power from energy available in its working environment. With self-powered biomedical devices, vital sign parameters can be continuously monitored and biomedical implants can be continuously powered, which reduce the risk of regular surgery. Self-powered systems help to achieve remote healthcare without exogenous power requirements. They are a great opportunity for developing countries since there is a big challenge regarding a continuous power supply and a lack of experienced scientists.

**Figure 5:**
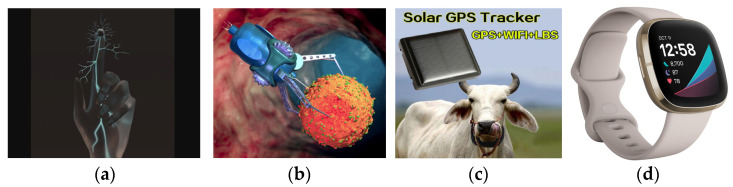
Future directions of self-powered biomedical devices in Africa: restoring the sense of touch to an injured finger (**a**) [83], intravenous drug delivery (**b**) [84], a self-powered GPS tracker for cattle (**c**) [85], and an e-health watch for temperature and heartbeat rate monitoring (**d**) [86].

Self-powered technology can enable the restoration of a sense of touch to injured or disabled patients (Figure 5a); they are great opportunity for medical care in inaccessible locations, with applications such as intravenous drug delivery by nanobots (Figure 5b), animal tracking devices (Figure 5c), monitoring and detection of early stage of non-communicable diseases such as CADs, diabetes, Alzheimer’s, etc., and continuous monitoring of remote patients living with critical health conditions (Figure 5d).

### 3.3. Status of Self-Powered Biomedical Devices in Africa

Biomedical research has shown its capability for stimulating the development of healthcare and biomedical infrastructures. In this section, we discuss the research status of self-powered devices in an African context and how research funding and scientific cooperation can provide opportunities to pursue this research area with regard to the populations in rural areas and developing countries.

The biomedical research field has reported significant achievements for healthcare devices in low- and middle-income countries, especially in African countries with the development of biomedical devices such as A blood glucose meter in Africa for Africans [16], a low-cost, non-invasive smart glucose monitoring device in South Africa [17], a free play fetal heart rate monitor [18], the SINAPI chest drain [19], a biomedical smart jacket [52], a vital sign monitor for expectant mothers [53], electronically controlled gravity feed infusion [54], the CARDIOPAD device for monitoring heart rate and forwarding data to remote scientist [14], a multi-function neonatal incubator for low- and middle-income countries [15], etc.

However, the research and development of self-powered devices is still at its stage of infancy. The reason behind this is that many funding institutions have restricted their support for solving specific health issues that affect the majority of a local population.

There are many financial institutions such as the African engineering award, the AFD (French Development Agency), the United Nation’s Development Program, the African Development Bank, Team Europe Initiative, etc.

Besides financial institutions, collaborative programs for biomedical research have greatly contributed to this research field, such as the U.S.–South Africa Program for Collaborative Biomedical Research (R01), the World Health Organization’s EU–Africa partnership, etc., to boost biomedical research. However, the biomedical research field in many low-income countries still faces serious challenges such as limited infrastructures and inaccessibility to biomedical research funding. In fact, the available funds for biomedical research aim to support only existing projects.

**Figure 6:**
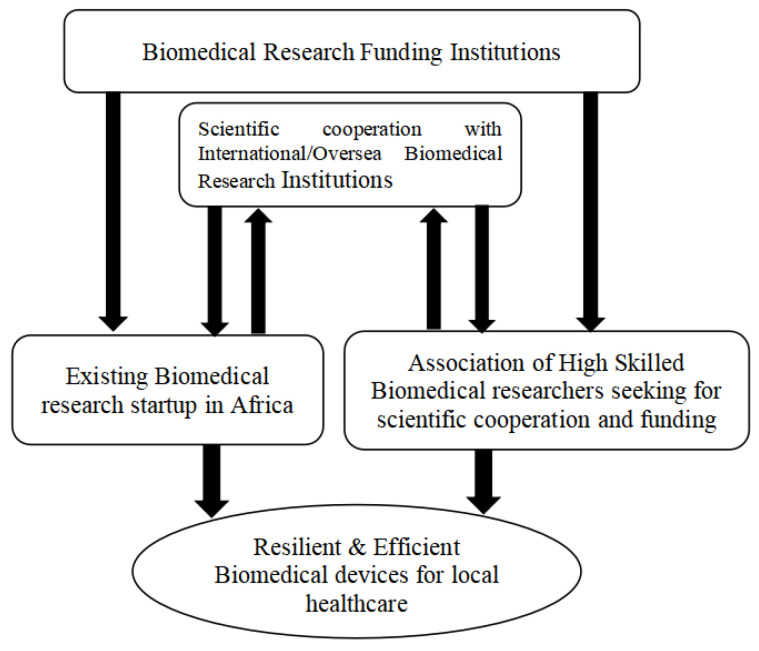
Architecture of scientific cooperation and funding in biomedical research suitable for low-and middle-income countries.

Scientific cooperation in the biomedical research field is restricted to biomedical research startups as well. Many biomedical research projects led by highly skilled researchers often fail due to lack of funding. Extending the research cooperation and funding to highly skilled graduate biomedical engineers who have no sufficient funds for realize their experimental prototype and start up a biomedical engineering company will help to lift the barrier in biomedical engineering research. Figure 6. illustrates the architecture of scientific cooperation and funding in biomedical research suitable for Africa and other middle-income country contexts.

For this cooperation and funding, both existing biomedical research startups and other highly skilled biomedical researcher groups can benefit from biomedical research funding and scientific cooperation with international research institutions.

## 4. Conclusions

This is the end of the current review which aimed to introduce the applications of self-powered biomedical devices, assess the status of the biomedical engineering research field in Africa and identify the challenges and opportunities. Many application areas of self-powered biomedical devices have been presented, including self-powered pacemakers, self-powered monitoring devices, non-invasive self-powered glucose meters, etc. The research status of biomedical devices in Africa has also been reported, including the existing achievements for biomedical devices and available scientific cooperation and funding for supporting biomedical research in Africa. However, some challenges to the development of biomedical research in Africa have been highlighted, including insufficient digital literacy training, absence or incompatibility of data for testing biomedical device algorithms, inaccessibility of infrastructures, restricted scientific cooperation and funding, absence of AI regulations, lack of government support and insufficient AI talent. An architecture has been provided for the better management of scientific cooperation and funding for biomedical engineering research suitable in an African context, which can enable the achievement of resilient and efficient biomedical devices for healthcare in Africa.

## Figures and Tables

**Table 1:** Definitions of self-powered devices.

Organizations	Definitions
Cambridge dictionary	Describes machines, etc., that do not need an outside energy supply to work as they have their own source of energy [2].
FORCE TECHNOLOGY	A self-powered system that charges itself with energy from its surroundings; it is a service-free solution. Depending on the surroundings, energy can be harvested from various sources, such as light, movement, heat, and magnetic and electrical fields [3].

**Table 2:** Contribution of biomedical energy harvesting TO self-powered healthcare devices.

Included Articles	Problem Being Solved	Solution	Research Question Answered	
[25]	Limited lifetime of battery technology for implantable devices, regular surgery for battery replacement for implants	Sub-dermal solar cell area under human skin for harvesting solar energy for powering wearable electronics	Applications of self-powered biomedical devices for healthcare	Implantable biomedical devices
[26]	Surgery is required for replacing the batteries of biomedical implants; late detection of cardiovascular diseases	Self-powered in vivo heartbeat rate monitoring device and real time transmission through Wi-Fi		
[27]	Limited lifetime of battery technology for implantable devices	A self-tuning inductive powering system		
[28]	Limited lifetime of battery technology for implants; lead-based ceramic piezoelectric nanogenerators are toxic and susceptible to fatigue cracking.	Self-powered cardiac pacemaker by a piezoelectric polymer nanogenerator implant		
[29]	Limited lifetime of battery technology for implants; hypertension disease requires continuous and accurate measurement of blood pressure.	Implantable and self-powered blood pressure monitoring based on a piezoelectric thin film		
[30]	Limited lifetime of battery technology for implants; many people suffer from CADs worldwide.	In vivo self-powered wireless cardiac monitoring via an implantable triboelectric nanogenerator		
[31]	Power requirement for biomedical devices; non-communicable diseases such as diabetes and CADs affect the majority of the population.	A battery-less implantable glucose sensor based on electrical impedance spectroscopy		
[32]	Biomedical devices are not compatible with living tissues and organs; power requirement for biomedical devices.	Biocompatible battery for medical implant charging via ultrasound		
[33]	Energy-harvesting systems cannot supply enough power to deep brain stimulation devices; damaged nerves do not allow the rest of the body to communicate with the brain.	Self-powered deep brain stimulation via a flexible PIMNT energy harvester, which harvests energy from cyclic deformations from heart, lungs, muscle, joints for stimulating brain and inducing behavioral changes.		
[34]	Many people worldwide suffer from osteoporosis and osteoporosis-related fractures; electrical stimulation requires external power, making it hard to miniaturize the device and improve portability	Self-powered implantable electrical stimulator for osteoblast proliferation and differentiation		
[35]	Power requirement for long-term animal monitoring devices	An implantable biomedical energy harvester for animal monitoring devices		
[36]	Existing non-invasive glucose detectors have the common problems of low portability, wearability and integrability	Self-powered triboelectric sensor for non-invasive glucose monitoring in human sweat		Non-invasive biomedical devices
[37]	Typical glucose sensors require an additional power supply and equipment for assessing glucose concentration	Flexible, disposable and portable self-powered glucose biosensors visible to the naked eye		
[38]	The energy generated by wearable bio-fuel cells is insufficient for powering read-out systems and communication protocols	Resettable sweat-powered wearable biosensor		
[39]	Many skin-path wearable sensors are limited by their dependence on silicon-based electronics, which increases the complexity and unit cost	Self-powered skin-path electrochromic biosensor		
[40]	Mass manufacture of electrochromic materials is limited by the need for transparent electrodes and liquid electrode systems	Fully printed and silicon-free self-powered electrochromic biosensors		
[41]	Limited lifetime of battery technology for biomedical devices, most wearable energy harvesters suffer from complex fabrication procedures, low power density, which make them unsuitable for biosensing	Wireless free-battery wearable sweat sensor powered by human motion, which extracts energy from a flexible printed circuit board (FPCB) based on a freestanding triboelectric generation process		
[42]	Biomechanical energy harvesting devices such as electromagnetic, piezoelectric and triboelectric energy harvesters have complicated structures, high production/maintenance costs and wearability and implantable site limitations.	Body-integrated self-powered system for wearable and implantable applications, which harvests energy through an electrode attached to skin for powering biomedical devices		
[43]	Issues in real-time detection of imbalances for patients requiring an immediate decision	Behavioral and environmental sensing and intervention (BESI), a sensor detecting extreme agitation in people with dementia		
[44]	Low glucose levels can lead to hypoglycemia, which has grave consequences for diabetics. Limited lifetimes of battery technology for biomedical devices	Detection of low glucose levels in sweat with colorimetric wearable sensors, which use a wearable colorimetric biosensor measuring glucose levels in sweat. The camera of a smart phone is then used for signal reading.		
[45]	Design and manufacturing of a soft body area sensor network relies on sophisticated approaches such as lithography or direct printing on carrier substrate before attaching to the body	Wearable circuits sintered at 3/4 room temperature directly on the skin surface for health monitoring, metal nanoparticles are printed on paper (fabric for flexible printed circuit boards), and sintered at room temperature and directly on human skin for on-body sensor with a novel sintering aid layer. Surface roughness is reduced and electromechanical performances are enhanced.		
[42]	Limited lifetime of battery technology for biomedical devices	Flexible wearable self-powered electronics based on a body integrated self-powered system (BISS), which uses behavioral and environmental sensing intervention for detecting early agitation of patients with Parkinson’s disease and sclerosis hyperplasia.		
[43]	Late detection of cerebral palsy, Parkinsons and multiple sclerosis diseases	Technology-Enabled Medical Precision Observation (TEMPO), which detects and records motion and provides healthcare with more accurate data for treating elderly patients and those with cerebral palsy, Parkinson’s and multiple sclerosis.		
[46]	Micro-super capacitors are promising alternatives for replacing lithium-ion batteries in wearable electronics, but they have a limited power density and a limited mechanical stretchability	High-energy all-in-one stretchable micro-supercapacitor arrays based on 3D laser-induced graphene foams decorated with mesoporous ZnP nanosheets for self-powered stretchable systems, which exhibit excellent ionic and electrical conductivity and impressive gravimetric capacitance and long-term stability.		
[16]	Diabetes management is expensive, and there is a need for self-monitoring of glucose levels at an affordable price with locally available materials in Africa	A blood glucose meter in Africa for Africans, which has an audio-visual output and a computer interface. It is made of microcontroller PIC16F877A for interfacing between a glucose sensor and an audio-visual unit (LCD and audio speaker). The glucose sensor is an electrochemical diagnostic strip, which uses glucose oxidase enzymes in conjunction with three electronically conductive electrodes. The chemical reaction produces a voltage. The voltage is processed by microcontroller using analog–digital conversion. Digital data are used to assess glucose levels and the results are sent to an audio-visual unit	Other biomedical device achievements in Africa	Nigeria
[47]	Permanent pacemaker implants encounter immediate post-procedure complications, including pneumothorax, hemothorax, air embolism, cardiac perforation etc.	Early experience with permanent pacemaker implantation at a tertiary hospital in Nigeria		
[48]	Cardiovascular disease affects the large majority of the population in the world and South Nigeria has an increased demand for invasive cardiac procedures, which are largely unavailable	Cardiac pacemaker insertion in the South of Nigeria: Prospects and challenges		
[17]	Prevalence of diabetes increases in middle- and low-income countries	Low-cost, non-invasive smart glucose monitoring device made in South Africa, which consist of three main parts: a transmitter (light source), a receiver (photo diode) and a processor (PIC16F877A), along with a data display section. Near-infrared is applied on the ear lobe, and the receiver receives the attenuated signal. The attenuated signal is used for assessing glucose level and is displayed on an LCD screen.		South Africa
[18]	A child is 500 times more likely to die during the first day of life than at one month of age in the developing world. Newborn mortality accounts for nearly 60 percent of infant deaths; biomedical devices designed for use in controlled sanitary conditions of first world hospitals do not stand a chance in rural Africa due to the harsh user environment and lack of proper training.	Free play fetal heart rate monitor, which measures the infant’s heart rate during birth and determines if the child is getting enough oxygen in the mother’s placenta. A low fetal heartbeat rate lowers the need for oxygen.		
[19]	Difficulties for evacuation of liquids and air from the chest, difficult patient management during mobility loss	SINAPI chest drain, which is custom-made, used post-cardiac surgery patients and fitted with a tube roller and a high gravity vent. The tube roller facilitates stripping of the tubing to remove clots, maintaining potency.		
[49]	Safety and effectiveness concerns over biomedical devices developed in Uganda	Formalize and establish a regulatory framework in Uganda for biomedical device developers		Uganda
[50]	27,000 children in Uganda die every year due to pneumonia; incorrect pneumonia diagnosis can be fatal	Biomedical smart jacket (Mama-Ope), which gives hope to mothers. It is a jacket measuring body temperature, heart rate and lung conditions. The jacket stretches across the whole chest and side of a patient. It monitors specific points on the lungs for symptoms of pneumonia. The jacket is connected to a smart phone via Bluetooth, which sends, records and analyzes the medical data, enabling healthcare professionals to make an informed diagnosis.		
[51]	Difficulty in diagnosis of fetal vital signs	Vital sign monitor for expectant mothers		
[52]	Many diseases require intravenous (IV) infusion therapy, but uncontrolled infusion rates and incorrect dosing can lead to severe complications or even death	Electronically controlled gravity feed infusion device, which monitors and controls drops with an accuracy of +/−10%. It has four main parts: -A microprocessor/logical unit, which receives inputs from peripherals, processes them and sends commands to actuators; -A drop rate detector module, which has a light source and a photocell signal for drop rate detection and transmits data to a micro-processor; -A user interface for human–machine interaction		
[14]	Unavailability of experienced scientists in rural areas for heart rate monitoring and medical advice	CARDIOPAD device for assessing heart rate and forwarding data to remote scientist through wireless communication		Cameroon
[53]	Cardiovascular diseases are emerging threats for the health of the population in Africa; 60% of the population in Africa live in rural areas and have no accessibility to appropriate healthcare	CARDIOPAD device, which is a small tablet for recording heartbeat rates and forwarding data by email to a remote scientist or cardiology hospital. The device can also directly generate a PDF file or send the data via Bluetooth transmission		
[15]	Over 8 million babies die prematurely in low-income countries	Multi-function neonatal incubator for low-income countries		
[54]	Cardiovascular diseases are constantly increasing worldwide, especially in low-income countries	Long-term prognosis of patients with permanent cardiac pacemakers in three cardiac centers in Cameroon		
[55]	The study of heart electrical conduction systems is limited only to mathematical modeling	Theoretical and experimental study of non-linear dynamics of a cardiac electrical conduction system		
[56]	Citizens with cardiovascular-related diseases are exposed to poor health service; the cost of acquiring healthcare-related technologies is high	Low-cost IoT-based remote cardiovascular patient monitoring system in Cameroon		

**Table 3:** Comparison of biomedical devices powered by Nanogenerators.

	Non-Invasive Biomedical Devices				In Vivo Biomedical Devices			
Source Type	Typical Application	Authors	Size	Harvesting Performance	Typical Application	Authors	Size	Harvesting Performance (Voltage, Power)
Electromagnetic energy generation/hybrid energy generation	Self-powered e-watch based on an electromagnetic triboelectric energy harvester	Teng Quan et al., Reproduced with permission from [57]	3.6 × 3.6 × 3 cm^2^	1.1 V 6.1 mW 0.35 mA	Miniaturized electromechanical devices for the characterization of the biomechanics of deep tissue	Enming Song et al. [58]	18 × 18 × 2.5 mm^2^	(50 Hz, 5 V)
	Electromagnetic triboelectric harvester for wearable electronics	Kewei Zhang et al. [59]	5 × 5 × 2.5 cm^3^ 60 g	4.9 mW and 3.5 mW 5.1 W/m^2^ 4.3 V 1.3 mA	Miniaturized EMEH for leadless cardiac pacemaker	Nicolas Franzina et al. [60]	Length 30 mm, Diameter 7 mm, Volume 1.15 cm^3^, mass 8.01 g	7.2 µW 200 mV
	Working Mechanism, Advantages and Limitations	Mechanism: Electromagnetic energy results from the motion of a coil through a stationary field Advantages: Suitable for harvesting energy from translational or rotational motions. Limitations: Hard to achieve design miniaturization; low power density compared to piezoelectric, triboelectric and photovoltaic energy generation.						
Piezoelectric energy generation	Piezoelectric BaTiO_3_ nanoparticles for biomolecule detection	Sophia Selvar ajan et al. [61]	7.5 × 1.5 × 1.6 cm^3^	60 mV 0.4 nW 0.022 nW cm^−3^	Piezoelectric nanogenerator for pace makers	Azimi et al. [28]	6.5 mm × 3.5 mm × 150 µm	6.06 V 143 µW/cm^3^
	A Shoe-Embedded Piezoelectric Energy Harvester for Wearable Sensors	Jingjing Zhao and Zheng You [62]	80 mm × 50 mm	3.6 V 4 mW	Experimental study on a piezoelectric vibration energy harvester for self-powered cardiac pacemakers	Feng Xie et al. [63]	6 × 2 × 0.1 mm^3^	3.5 mV 60 nA
	Working Mechanism, Advantages and Limitations	Mechanism: Piezoelectricity is generated by applying mechanical stress to a piezoelectric material. Advantages: Suitable for harvesting strain or pressure energy, higher power density compared to electromagnetic energy harvesting, possibility to miniaturize the design, suitable for in vivo biomedical applications. Limitations: Low power density compared to compared to triboelectric and photovoltaic energy generation.						
Triboelectric energy generation	Triboelectric nanogenerators for self-powered sensing	Yaojie Han et al. [64]	5 × 5 cm^2^	0.35 µA 130 V 45.8 µW cm^−2^	Self-powered energy harvesting and implantable storage system based on hydrogel-enabled all-solid-state supercapacitor and triboelectric nanogenerator	Zhuo Wang et al. [65]	2 × 4 cm^2^	95.04 V 1.38 µA 9.03 µW cm^−2^
	A triboelectric nanogenerator as a self-powered temperature sensor based on PVDF and PTFE	Kequan Xia et al. [66]	60 cm × 3 cm × 1 mm	49 V 240 µW	Self-rechargeable cardiac pacemaker system with triboelectric nanogenerators	Hanjun Ryu et al. [10]	Radius 1.5 cm Height 2.4 mm	4 V 4.9 µW cm^3^
	Working Mechanism, Advantages and Limitations	Mechanism: Triboelectric energy results from friction between two different triboelectric materials. Advantages: Higher power density compared to electromagnetic and piezoelectric energy harvesting, possibility to miniaturize the design, suitable for friction energy harvesting, suitable for in vivo biomedical applications. Limitations: The structure of triboelectric nanogenerator needs to retain a small gap for contact separation, so further efforts are needed for miniaturizing the design. Miniaturizing the design affects the output performance. Finding the balance between size and power performance is challenging for biomedical applications.						
Photovoltaic energy generation	Flexible-fabric-based GaAs thin-film solar cell for wearable energy harvesting applications	Yeojun Yun et al. [67]	0.2 cm^2^	0.972 V 100 mW/cm^2^ 22.59 mA/cm^2^	Photovoltaic Power Harvesting Technologies in Biomedical Implantable Devices Considering the Optimal Location	Jinwei Zhao et al. [68]	P+ Layer 1 × 1020 cm^−3^ Layer 4.6 × 1015 cm^−3^ Layer 1 × 1016 cm^−3^	0.675 V 17.20 mW 100 mW/cm^2^ 31.42 mA/cm^2^
	Solar and Thermal Energy Harvesting with a Wearable Jacket	Quinn Brogan et al. [69]	31 × 31 × 3.0 (L × W × D) mm	Open circuit voltage: 2.2 V per cell Peak voltage: 1.2 V per cell 475–500 mW	Energy Harvesting by Subcutaneous Solar Cells: A Long-Term Study on Achievable Energy Output	L. BEREUTER et al. [70]	3.6 cm^2^	s 67 µW (=19 µW cm^−2^)
	Working Mechanism, Advantages and Limitations	Mechanism: Sunlight hits solar cells and electrons in the cells are energized, start moving and then flow out of the junction between cells layers, creating electric current. Advantages: Highest power density compared to electromagnetic, piezoelectric and triboelectric energy generation. Limitations: Not applicable for in vivo biomedical energy harvesting since solar cells need to be exposed to sunlight.						
